# Pathogenicity and virulence of *Erwinia amylovora*

**DOI:** 10.1080/21505594.2026.2664987

**Published:** 2026-04-26

**Authors:** George W. Sundin, Luisa F. Castiblanco, Quan Zeng

**Affiliations:** a Department of Plant, Soil, and Microbial Sciences, Michigan State University, East Lansing, MI, USA; b Department of Plant Pathology & Ecology, The Connecticut Agricultural Experiment Station, New Haven, CT, USA

**Keywords:** Fire blight, type III effector, biofilms, apple, host resistance, disease management

## Abstract

Fire blight, caused by the bacterial pathogen *Erwinia amylovora*, is the most significant global threat to commercial apple and pear production. Outbreaks resulting in flower infection cause yield losses accompanied by internal systemic spread of pathogen cells through trees that can lead to tree death in a single season. The type III secretion system and the exopolysaccharide (EPS) amylovoran are pathogenicity factors, and biofilm formation in leaf xylem is a critical virulence factor. We review our current knowledge of *E. amylovora* pathogenesis and how pathogenesis strategies intersect and facilitate systemic movement in the host. Fire blight management remains extremely difficult due to the high susceptibility of host plants and the aggressive virulence of the pathogen. We suggest that future management will rely on innovative strategies for resistance breeding or those that directly target bacterial populations and condition the host by eliciting a resistance response prior to pathogen arrival.

## Introduction

*Erwinia amylovora* is the causal agent of fire blight disease of woody plants in the Rosaceae family and occurs in commercial apple and pear orchards in all-growing regions of the world except for Australia and South America. Fire blight symptoms develop rapidly following infection, resulting in rapid killing of flower clusters and branches. Branch (shoot blight) infections are characterized by rapid leaf browning and the “shepherd’s crook” curvature of infected shoots, giving affected trees a scorched appearance from which the disease derives its name. Fire blight epidemics can escalate quickly because *E. amylovora* cells are readily dispersed by wind, rain, and insect activity [1,2]. Damaging storms with hail and high winds can result in large outbreaks that appear suddenly. Economic losses due to fire blight and the cost of management are estimated to exceed $100 million annually in the United States. Notable epidemics include those in Michigan in 2000 and New York in 2016, with the estimated losses of $42 million and $16 million, respectively [3–5]. Internationally, fire blight has prompted drastic management responses, exemplified by large-scale eradication efforts in South Korea, where 798 ha of trees representing 63.1% of orchards in one growing region were removed and buried in 2019–2020 [6].

Modern fruit tree breeding has historically prioritized fruit size, yield, and quality. It is apparent that these horticultural traits are not linked with traits that confer reduced disease susceptibility to fire blight, as most of the commercially successful apple, pear, and Asian pear cultivars grown in the 2020s are highly susceptible to fire blight. Modern tree fruit production is also trending toward growing trees in high-density plantings with dwarf trees trained to narrow vertical architectures. This production strategy, although highly efficient for fruit production, can also greatly facilitate the spread of fire blight within and between trees, fueling disease epidemics and risk of major losses worldwide. Global commerce has further facilitated fire blight spread into previously unaffected geographic locations, likely through the movement of nursery stock. Since the mid-2000s, fire blight has spread into Central Asia, including Kazakhstan and Kyrgyzstan [7], and more recently into South Korea and China during the 2010s [8,9].

Thus, although fire blight disease has been known for over 230 y, management remains extremely difficult due to the high susceptibility of host plants and the aggressive virulence of the pathogen. Here, we will review the biology and disease cycle of the pathogen, discuss the phylogeny of *E. amylovora* and molecular subtyping of strains, dissect the molecular biology of host–pathogen interactions, and cover recent advances in disease management.

## Disease cycle, flower, branch, and rootstock infection

**Figure 1.**
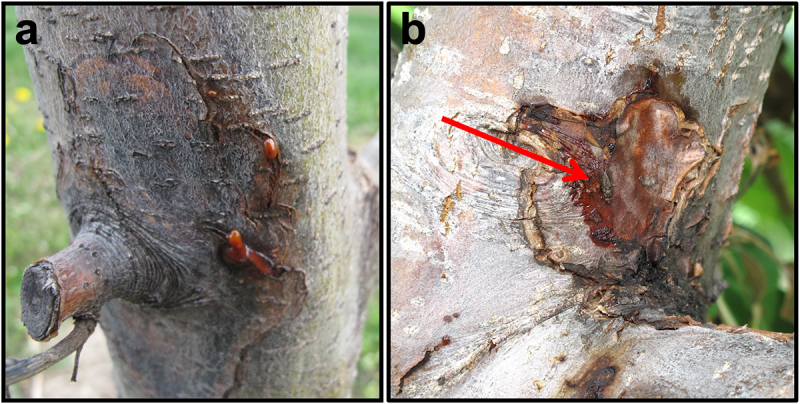
(a) Fire blight canker on the main trunk of a “Ginger gold” apple tree in spring. Two *Erwinia amylovora* red ooze droplets have emerged on the canker surface. (b) Fire blight canker on the main trunk of a “Jonathan” apple tree in spring with ooze exudates on the surface. The red arrow denotes a fly that has landed on the ooze and can potentially transfer *E. amylovora* cells to nearby flowers.

*Erwinia amylovora* cells are associated year-round with their plant hosts. During winter, bacterial cells overwinter in cankers (Figure 1(a)), localized dead lesions in woody tissue, serve as the primary inoculum source for the next season [10]. Warming temperatures in the spring stimulate the exudation of *E. amylovora* cells in droplets of bacterial ooze that appear on the canker surface. Ooze contains highly concentrated populations of *E. amylovora* (10^8^−10^9^ cells μl^−1^) and the exopolysaccharide amylovoran [11,12]. The dark color and sugar content of *E. amylovora* ooze is attractive to insects, such as bees and flies, which visit the ooze droplet and readily acquire bacterial cells on their legs and body (Figure 1(b)). These flies can then carry *E. amylovora* cells to nearby open flowers, depositing cells onto flower stigmas [2,13]. Stigmas function in the capture of pollen grains and provide a conduit to the flower ovary via a stalk structure termed the style. Apple and pear flower stigmas are comprised of elongated cylindrical papillae cells that function in the capture of pollen grains for fertilization and fruit development [14,15]. The intercellular space at the base of these papillae cells harbors exudates containing sugars such as glucose and fructose and amino acids [16]. In addition, arabinogalactan proteins are secreted at stigma tips and are crucial for stigma receptivity to pollen germination [14].

**Figure 2.**
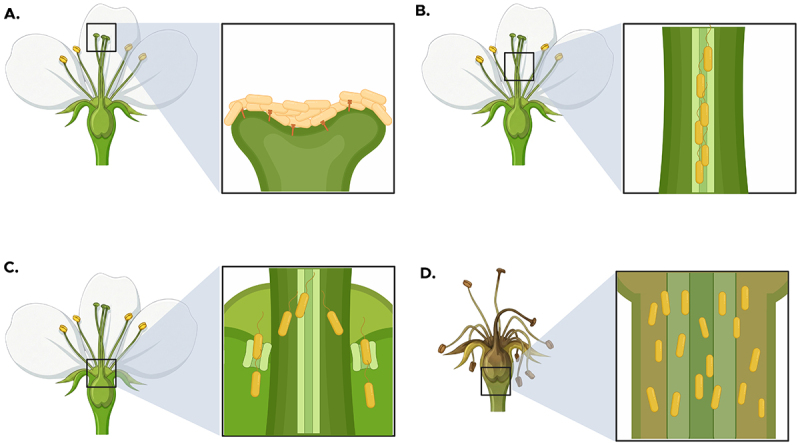
Flower infection process during fire blight pathogenesis. (a) *E. amylovora* grows epiphytically in the intercellular spaces of the papillae cells of the stigmas, where a subpopulation employs its *hrp*-T3SS to translocate effector proteins, promoting bacterial multiplication in the stigma surface. (b) Directed by flagellar-mediated chemotaxis to organic acids found in nectar, *E. amylovora* migrates down the stylar groove from the stigma to the hypanthium. (c) Upon reaching the base of the flower, the pathogen invades the nectaries, which serve as natural openings for nectar secretion, and (d) continues its systemic invasion of the host, leading to the development of blossom blight. Figure created in https://biorender.com.

**Figure 3.**
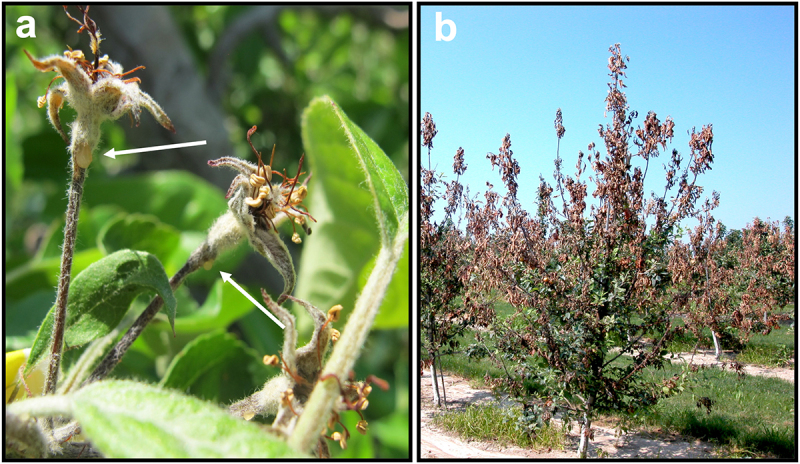
(a) “Gala” apple flowers exhibiting blossom blight symptoms. Systemic migration of *E. amylovora* cells occurs downward through the flower pedicel (arrows denote ooze droplets) as the pathogen population is partitioned to cells migrating through the host or functioning in dispersal. (b) Shoot blight symptoms on a blighted “Gala” apple tree in a commercial orchard in Michigan, USA.

The stigma is the critical habitat where *E. amylovora* cells grow to large populations prior to flower infection [17,18]. Flowers that are open from 1 to 3 d are the most receptive for *E. amylovora* growth, and cell populations can increase as much as 10^4^-fold d^−1^ with as many as 10–15 cell generations within a 24-h period [19] (Figure 2(a)). Populations can exceed 10^6^ cells per flower, and field studies indicate that populations exceeding 10^4^ cells per flower are required to cause flower infection and blossom blight symptoms [1]. Pollinating insects such as honeybees (*Apis mellifera*) and bumble bees (*Bombus impatiens*) also efficiently spread *E. amylovora* from colonized to healthy flowers and can play a significant role in larger disease outbreaks [2,20]. While *E. amylovora* can utilize all the reported stigma exudate sugars and amino acids for growth [16], it has also recently been shown that *E. amylovora* can metabolize arabinogalactan, a trait that facilitates early competitive interactions with other microbes during initial colonization of stigmas [21]. Environmental conditions strongly influence bacterial growth on stigmas, with temperature, relative humidity, and low windspeed being the critical ones [19]. Notably, *E. amylovora* growth predominantly occurs at night, when low windspeed favor dew formation. Dew is postulated to provide free moisture and an influx of heat into the microenvironment of the stigma that stimulated bacterial growth [19]. As little as 0.25-mm rainfall is enough for *E. amylovora* to migrate down the style to the base of the flower (hypanthium) with flagellar motility [22] (Figure 2(b)). Scanning electron microscopy of the style of apple and pear flowers by Spinelli et al. [23] has shown the presence of a stylar groove or furrow that extends to the hypanthium. Further confocal microscopy analysis using *gfp*-labeled *E. amylovora* demonstrated that these cells migrate from the stigma to the hypanthium via the stylar groove [23]. Thus, this anatomical feature of apple and pear flowers has likely been co-opted by the *E. amylovora* pathogen to act like a “sliding board” for pathogen cells that are carried by water flow, influenced by gravity, to the hypanthium. Upon reaching the hypanthium, *E. amylovora* cells next navigate downward through floral nectar and enter plants through nectarthodes (natural openings that secrete nectar) leading into the floral nectaries where infection is established [24] (Figure 2(c)). Flower infection results in necrotic blossom blight symptoms (Figure 2(d)); from here, *E. amylovora* cells move systemically in the host from flower structures into branches and can also emerge in ooze droplets (Figure 3(a)) that facilitate the transfer of cells via wind, rain, and insect activity, to new infection sites both within and between trees.

Infection of branches (shoots) by *E. amylovora* cells occurs via developing leaves at shoot tips. Entry points appear mainly to be wounds caused by weather-induced mechanical injuries, such as hail damage, insect feeding, or from wounds generated by the abscission of trichomes [1,25]. Shoot infections result in the classic shepherd’s crook appearance of a wilted, dying branch (Figure 3(b)). Shoot infections can be initiated from as few as 100 cells [26] and are characterized by rapid pathogen growth. For example, inoculation experiments conducted under field conditions demonstrated growth from 10^5^ cfu g^−1^ of tissue to >10^9^ cfu g^−1^ in 7 d in leaves at shoot tips and from 10^3^ cfu g^−1^ of tissue to >10^9^ cfu g^−1^ in 5 d in stem tissue further down the branch [27]. *E. amylovora* cells in infected shoots can continue to spread downward to the trunk of the tree and further to the rootstock crown. In highly susceptible apple cultivars, this downward spread can occur within a single season or over multiple seasons, culminating in the formation of a girdling canker at the base of the tree or in the rootstock that results in tree death.

## Importance of fire blight disease to commercial apple and pear production

Phylogenetic evidence suggests that the *E. amylovora* evolved in North America on native crabapple species, such as *Malus coronaria* [28]. In contrast, modern cultivated apples are descendants of *M. sieversii*, which evolved in Central Asia [29] and therefore did not co-evolve with *E. amylovora*. In plants, the lack of coevolution of a host:pathogen pair often translates into limited host resistance or tolerance, increasing the likelihood of severe disease outbreaks when the pathogen is introduced into a new geographic location. For example, similar patterns of severe disease emergence have occurred with invasive pathogens, such as those causing Dutch elm disease and chestnut blight in North America, which are characterized by rapid epidemic spread and persistence of the introduced pathogen [30,31]. Fire blight disease has arisen similarly, but is an invasive disease in reverse, as the pathogen-naive host was brought to North America by European settlers in the 1600s [28]. Fire blight was first observed in 1780 in the Hudson Valley of New York and first reported in 1794 [32].

## Phylogeny of *E. amylovora*

**Figure 4.**
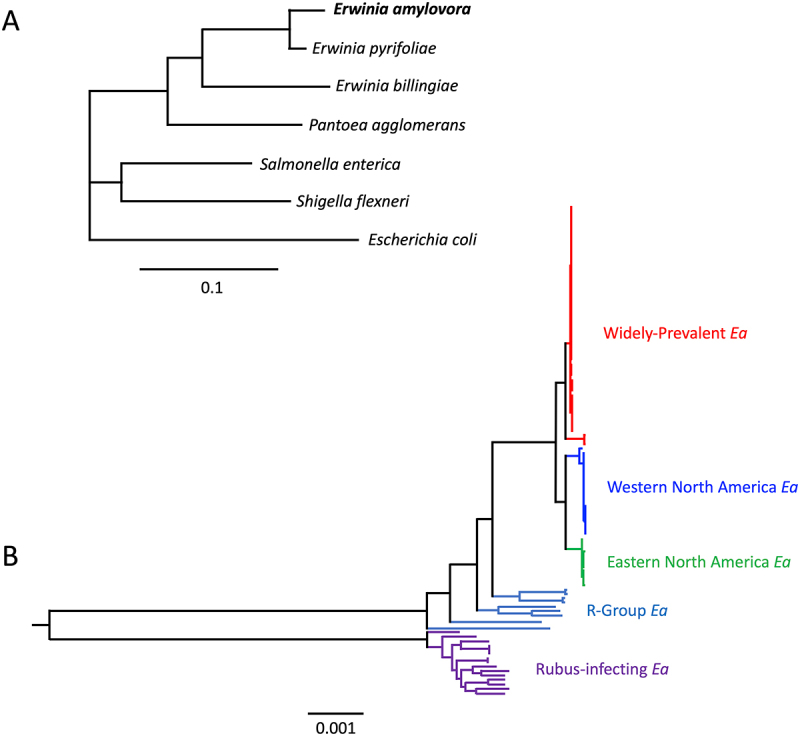
Phylogenetic relationships of *Erwinia amylovora* relative to other human enteric pathogens (A) and among *E. amylovora* strains (B). Panel (A) was generated using 16S rRNA sequence alignments of the respective bacterial taxa, while panel (B) was constructed using a concatenated chromosomal alignment and a maximum-likelihood phylogenetic model. *E. amylovora* strains are color-coded according to their assigned clades.

*Erwinia amylovora*, formerly a member of the family *Enterobacteriaceae*, is now a member of the family *Erwiniaceae* in the order *Enterobacterales* [33]. Within the order *Enterobacterales*, the *Erwiniaceae* family is most closely related to the family *Enterobacteriaceae*, which also includes well-known human and animal pathogens, such as *Escherichia coli*, *Salmonella enterica*, and *Shigella flexneri*. Consequently, from a genetic and evolutionary perspective, *E. amylovora* is more closely related to certain enteric pathogens than to many other plant-associated bacteria, such as *Pseudomonas syringae* (Figure 4(a)). Consistent with this relationship, *E. amylovora* employs virulence strategies that are also characteristic of enteric pathogens, including the delivery of effector proteins into host cells via the Type III secretion system and biofilm formation during host colonization [34,35].

Phylogenetic analyses group *E. amylovora* into three major superclades: the Amygdaloideae-infecting *E. amylovora* (causing fire blight on apple and pear), the *Rubus*-infecting *E. amylovora* (infecting raspberry), and the B-Group (Figure 4(b)) [36,37]. Among these superclades, the B-Group exhibits the highest intracladal diversity, with a pairwise nucleotide identity of 99.76%, followed by the *Rubus*-infecting *E. amylovora*, with a pairwise identity of 99.89% [36]. In contrast, the Amygdaloideae-infecting *E. amylovora* displays the lowest intracladal diversity with a pairwise identity of 99.93% [36]. Compared with other plant pathogens, the genetic diversity within the Amygdaloideae-infecting *E. amylovora* is exceptionally low. The Watterson theta (*θ_w_*) for this group is 1.87^−4^ per site, whereas the θ_w_ values of *Pseudomonas syringae* pv. *tomato* and *P. syringae* pv. *kiwi* are 6.92^−3^ and 8.00^−3^ per site, respectively. Notably, these highly homogeneous *Amygdaloideae*-infecting strains were isolated across wide geographic regions over a 57-y period, suggesting that host selection, rather than environmental factors, is the primary driver for the genetic diversification within *E. amylovora* [36]. Despite this remarkable genetic homogeneity, *E. amylovora* isolates display substantial variation in aggressiveness and symptom severity on host plants [37,38]. In some cases, a single nucleotide polymorphism, such as one affecting the global virulence regulator Hfq, is sufficient to cause pronounced differences in virulence phenotypes [37]. Large chromosomal inversions have also been identified among *E. amylovora* genomes, although their contribution to strain aggressiveness remains unclear [39,40]. Further studies are therefore required to elucidate the genetic mechanisms underlying these phenotypic differences.

The *Amygdaloideae*-infecting *E. amylovora* superclade can be further subdivided into three primary clades: the Widely–Prevalent clade, the Eastern North America clade, and the Western North America clade (Figure 4(b)) [36,37]. While isolates from Eastern and Western North America clades are geographically restricted to their respective regions, the Widely–Prevalent clade has been detected outside North America, including in Switzerland, New Zealand, Israel, and France. This clade exhibits a higher level of recombination with strains from the Eastern North America clade, suggesting that the Widely–Prevalent clade likely originated in the eastern United States before spreading globally [37].

## CRISPR genotyping for strain differentiation in *E. amylovora*

The homogeneity of the *E. amylovora* genome has led researchers to scour genomes for decades in attempts to find more diverse or faster evolving sequences that could be used for strain tracking from local to continental levels. A wide range of studies have been published on the genetic comparison of strains using RAPDs, Rep-PCR, AFLP, MLVA, short sequence repeats (SSRs) (reviewed in [41]). Methods capable of resolving high levels of diversity among the *E. amylovora* strains tested are particularly valuable for epidemiological studies, such as tracking strain movement, identifying sources of outbreaks, and assessing the abundance of genotypes within a particular location. Over the past 15 y, clustered regularly interspaced short palindromic repeats (CRISPRs) have emerged as the “gold standard” for comparative analyses of *E. amylovora*. CRISPR spacer arrays are one part of a bacterial immune system that mediates sequence-specific exclusion of invading DNA from bacteriophages, conjugative plasmids, and other sources [42]. The sequences of individual spacers, characteristically 32 bp in length, reflect the history and temporal exposure to foreign DNA that stimulated the immunity response, as new spacers are sequentially incorporated at the 5’ end of each array [43]. Spacer sequences also appear to have a geographic component, as foreign DNA invaders likely differ in different regions. CRISPR-based subtyping has become a critical molecular tool in public health for epidemiological studies, strain tracking and microbial outbreak tracing, and for isolate identification to enable rapid management responses [44]. CRISPR subtyping has become widely adopted for *E. amylovora* and is an important tool for microbial strain tracking and for understanding the evolution of *E. amylovora* at regional and global scales.

CRISPR studies with *E. amylovora* were initiated with two studies both evaluating global distributions of strains [45,46]. Both of these studies demonstrated that major strain groups could be identified and also that *E. amylovora* strains from different hosts such as *Rubus* spp. and loquat possessed distinct collections of spacer arrays [45,46]. CRISPR spacer patterns of United States strains readily differentiated strains isolated in the eastern United States from those isolated in the western United States, suggesting that strains from different sources were involved in colonizing apples and pears as European settlers brought these trees westward [46]. More recently, CRISPR analyses have been performed on more local scales, tracking strain introduction into new countries and regions and identifying new genotypes [47–51] and also for tracking of strains with streptomycin resistance [52–55]. In the future, combining knowledge of CRISPR genotypes with quantitative virulence phenotypes will provide important information on epidemiology and strain dispersal. The impact of specific control strategies and tactics on the population dynamics of specific *E. amylovora* (CRISPR) genotypes could also provide more precision in understanding the effectiveness of treatments for fire blight management.

## Molecular mechanisms of pathogenicity

*Erwinia amylovora* encodes two critical determinants, the type III secretion system (T3SS) and the exopolysaccharide (EPS) amylovoran that are absolutely required for pathogenesis on Rosaceae family woody hosts. We will summarize the current knowledge of the function and molecular regulation of both of these systems below. In addition to these major disease pathways, *E. amylovora* encodes numerous other genes that impact virulence including pathways for the uptake and utilization of sorbitol and sucrose, the two major storage and transport caarbohydrates in Rosaceae plants, the desferroxamine siderophore functioning in iron scavenging, multidrug efflux pumps that can export plant antimicrobial defense compounds, and the type VI secretion system. Information on these additional virulence determinants has been published previously [1,56].

### Type III secretion system and the type III effector DspE

Similar to other Gram-negative pathogenic bacteria, *E. amylovora* possesses a type III secretion system (T3SS), a specialized needle-like protein transport apparatus that translocate proteins, known as effector proteins, from the bacterial cytosol to the host cytoplasm or periplasm. These effectors hijack the host genetic machinery to promote bacterial survival and pathogenesis [57,58]. Genes encoding the structural and regulatory components of T3SSs are typically organized in large genomic clusters. In plant pathogenic bacteria, these clusters have been collectively referred to as *hrp* (*h*ypersensitive *r*esponse and *p*athogenicity) gene clusters, as mutations in some of these genes render the bacteria nonpathogenic in susceptible plants and unable to elicit an immunity-induced programmed cell death (hypersensitive response) in resistant plants [59,60].

The *hrp* gene cluster of *E. amylovora* is a central virulence determinant and resides within a 62-kb pathogenicity island (PAI1), which also includes an island transfer region suggestive of horizontal acquisition [61]. The cluster comprises 34 genes, including nine encoding structural core proteins, four encoding effector and chaperone proteins, and four regulatory genes [61]. Transcription of these genes is positively regulated by the alternate sigma factor HrpL, which binds to a conserved motif known as *hrp*-box, located in the promoter regions of target genes, and subsequently directs the RNA polymerase for transcription initiation [62,63]. Interestingly, the *hrp*-box sequence (5’-GGAAC-N_(16–20)_ACNNC-3’) has also been found in the promoter regions of genes unrelated to type III secretion, suggesting that these might play an important role in the infection and host colonization process [63].

**Figure 5.**
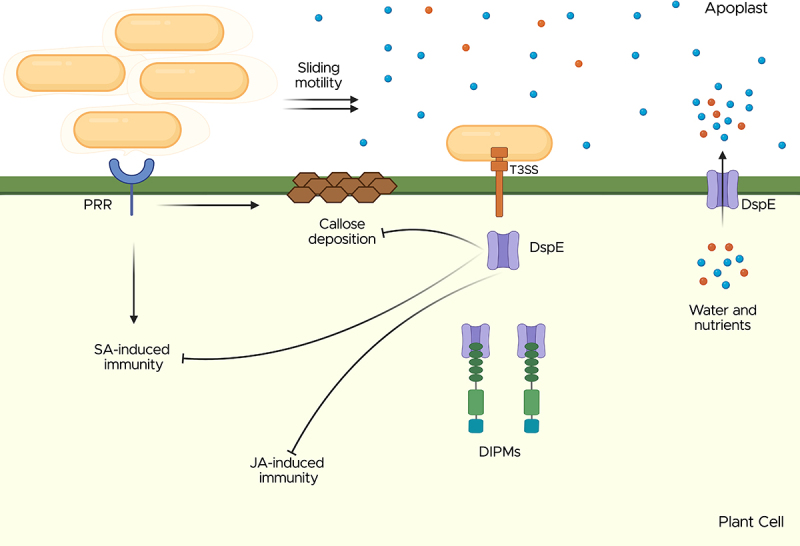
*E. amylovora* alters apoplastic water content and osmotic balance of infected plant cells through T3 translocation of DspE. *E. amylovora* employs its hrp-T3SS to secrete and translocate DspE into host plant cells, where DspE forms water- and solute-permeable channels. These channels contribute to the formation of a water-soaked and nutrient-rich apoplastic environment, that facilitates systemic movement of the pathogen throughout host tissues via exopolysaccharide (EPS)-driven sliding motility in a flagellar-independent manner. Additionally, DspE suppresses host immune responses triggered by the recognition of pathogen-associated molecular patterns (PAMPS), including callose deposition, SA-dependent immunity responses and JA-dependent signaling. This immune suppression is likely mediated through interactions with host proteins such as DIPMs (DspE-interaction proteins from *Malus domestica*) and PRR (PAMP-recognition receptors). Figure created in https://biorender.com.

Compared to other plant pathogenic bacteria, *E. amylovora* possesses a relatively small repertoire of effector proteins. DspA/E (referred to as DspE henceforth), one of the proteins encoded within the *hrp*-T3SS cluster, was the first effector protein identified in *E*. *amylovora*. DspE has been characterized as an essential pathogenicity factor for this pathogen, as its expression and delivery into the host cell are required for the development of symptoms and successful bacterial proliferation in the host [64–66]. DspE is a large multidomain protein belonging to the AvrE family of effectors, which includes several homologs from the genera *Erwinia*, *Pantoea*, *Dickeya*, *Pseudomonas*, and *Pectobacterium* [67,68]. Members of this family are involved in creating an aqueous environment in the plant apoplast conducive to bacterial multiplication, which leads to typical water-soaking symptoms in susceptible plants [69–71]. Nomura and collaborators [72] recently demonstrated that the C-terminal end of DspE in *E. amylovora*, as well as AvrE from *P. syringae*, folds in a β-barrel stem structure that forms a water and solute-permeable channel with a predicted pore size of 15–20 Å. These channels enable the pathogen to alter the apoplastic water content and osmotic balance of infected cells [72] (Figure 5).

In addition to its direct biological function, DspE is involved in the inhibition of defense response signaling. Boureau and colleagues [65] demonstrated that DspE contributes to the suppression of salicylic acid (SA)-dependent innate immunity, as it delays the expression of the host gene *PR1*, a SA-induced defense marker in plants, when transiently expressed in nonhost tobacco plants (Figure 5). Similarly, mutants lacking *dspE* activate callose deposition, a cell wall reinforcement that acts as a physical barrier against pathogens [73,74]. DspE also interferes with the expression of genes related to the jasmonic acid (JA)-mediated immunity response in susceptible apple plants [75] and alters intracellular trafficking and actin cytoskeleton architecture in yeast [76], indicating a global impact of this effector on host cellular processes. The N-terminal portion of DspE interacts with four proteins in apple, designated as DIPM 1–4 (DspE-interacting protein from *Malus* × *domestica*), which belong to the leucine-rich repeat (LRR) receptor-like serine/threonine kinases family of transmembrane proteins, known for playing an important role as components of defense related-signal transduction cascades, among other cellular processes in plants [77,78] (Figure 5). Silencing of these genes via interference RNA (RNAi) and CRISPR/Cas9 gene editing techniques significantly reduces host susceptibility to *E. amylovora* [79,80], indicating that DspE targets these proteins to suppress plant immunity.

### Other T3 effector proteins

Genomic and proteomic analyses have revealed that, in addition to DspE, *E. amylovora* secretes at least four other effector proteins via its hrp-T3SS: AvrRpt2_Ea_ (Eop4), HopPtoC_Ea_, Eop1, and Eop3 [35,81–83]. AvrRpt2 _Ea_ (also known as Eop4) shares 62% amino acid sequence identity with the well-characterized AvrRpt2 effector from *P. syringae* pv. *tomato*, which interferes with the PAMP-triggered immunity (PTI) signaling cascades and promotes virulence by stimulating the turnover of auxin/indole acetic acid proteins, thereby altering the host auxin physiology [84–86]. AvrRpt2_Ea_ is a cysteine protease and functions as a virulence factor in *E. amylovora*. Mutants lacking *avrRpt2*_*Ea*_ exhibit reduced virulence and bacterial multiplication when inoculated into immature pear fruit [83]. Consistent with this role, transient expression of *avrRpt2*_*Ea*_ in transgenic apple plants of a susceptible cultivar leads to the development of symptoms similar to those produced by *E. amylovora* in a natural fire blight infection, such as shoot necrosis, shoot tip wilting, and browning of leaves, as well as the induction of SA synthesis and expression of SA-responsive genes [87].

Eop1 (also referred to as EopB or OrfB) is a member of the YopJ/AvrRxv family of effector proteins, which are characterized by their cysteine protease and acetyltransferase activities [88]. Although the biological function of Eop1 has not been determined, Eop1 appears to act as a host specificity determinant, as the expression of *eop1* from a *Rubus* spp. infecting strain significantly reduced the virulence of *Spiraeoideae*-infecting *E. amylovora* strains in immature pears and apple shoots [89].

The effector proteins HopPtoC_Ea_ and Eop3 do not appear to play direct roles in the virulence of *E. amylovora*, as deletion of these genes did not affect the bacterial virulence compared with the wild-type strain, in immature pear infection assays [82,90]. However, transcriptomic analyses revealed that a highly virulent strain of *E. amylovora* expresses significantly higher levels of *eop3* than a low-virulence strain, both *in vitro* and *in planta* [91]. Interestingly, overexpression of *eop3* resulted in a significant reduction in symptom development relative to the wild-type strain in a fire blight susceptible apple cultivar, suggesting that Eop3 is an avirulence protein that is recognized by the host and subsequently triggers a defense response [90]. Moreover, the deletion of *eop3* resulted in the suppression of HR in *Nicotiana tabacum* but not in *N. bethamiana*, suggesting that this effector may interfere with the elicitation of programmed cell death when not recognized by a corresponding resistance protein [90].

Effector translocation in *E. amylovora* is facilitated by T3 chaperone proteins, which typically bind to a region in the N-terminal portion of their cognate effectors. T3 chaperones not only stabilize effectors, but also modulate the secretion hierarchy and specificity of the effector repertoire [92,93]. The translocation of DspE is facilitated by the T3 chaperone protein DspF (also referred to as DspF/B), by binding to the N-terminal region of the effector [66,94]. In addition, Castiblanco and colleagues [95] demonstrated that the cognate T3 chaperones of Eop1 and Eop3, Esc1, and Esc3, work cooperatively to promote the secretion and translocation of DspE. Notably, translocation of Eop1 and Eop3 is significantly increased in a *dspF*-mutant background, indicating that DspF negatively regulate their secretion and translocation and suggesting a role of DspF in the establishment of an effector export hierarchy within *E. amylovora* [95]. To date, no T3 chaperones have been identified for HopPtoC_Ea_ or AvrRpt2_Ea_.

### HrpN

In addition to effectors, the *hrp-*T3SS of *E. amylovora* secretes the harpin protein HrpN. Harpins are heat-stable, glycine-rich proteins produced exclusively by Gram-negative plant pathogenic bacteria and are defined by their ability to elicit a hypersensitive response when externally applied to plant cells [96]. HrpN, the first reported harpin, is required for full virulence in *E. amylovora* in host plants and is a key component of the translocator complexes for the translocation of effector proteins, as translocation of DspE was significantly reduced in an *hrpN*-mutant background [97–100]. Consistent with this function, HrpN has been shown to form ion-conducting pores in liposomes, suggesting that it facilitates effector translocation by inserting into the host cell plasma membrane and exhibiting a pore-forming activity [101]. In addition to its role as a facilitator of effector delivery, HrpN has been demonstrated to be translocated to the host cytoplasm as well, where it triggers the activation of defense responses, such as callose deposition and the massive production of reactive oxygen species (ROS) [102,103].

### Expression of T3SS genes

Expression of the T3SS in *E. amylovora* is tightly regulated in a spatially and temporally dependent manner, varying with plant tissue type, infection stage, and environmental conditions [104–106]. During flower infection, T3SS gene expression is highest at the early stages of colonization on the apple stigma, compared with expression in the hypanthium [104] or the pedicel [105]. Although T3SS activity is generally associated with post-entry interactions within host tissues, *E. amylovora* actively deploys a functional T3SS to translocate the effector DspE while still residing on the stigma surface [104]. A functional T3SS is essential for efficient stigma colonization. T3SS-deficient mutants of *E. amylovora* exhibit approximately an order of magnitude reduction in population size on apple stigmas, indicating that the pathogen actively disrupts host cells to release nutrients that support epiphytic proliferation, a capability largely absent in nonpathogenic commensal bacteria [104,107]. Furthermore, the stigma surface represents a T3SS-inducing environment, whereas the hypanthium, where bacterial entry into host tissues occurs, constitutes a T3SS-repressive environment. Growth on the stigma therefore preconditions *E. amylovora* to overcome T3SS repression in the hypanthium, facilitating successful blossom blight infection [104].

Despite the critical role of the T3SS in colonization and infection, only a subpopulation of *E. amylovora* cells actively expresses T3SS at any given time [104]. This heterogeneity may reflect an energy-conservation strategy, as individual plant cells are substantially larger than bacterial cells and can be contacted by tens to hundreds of bacteria simultaneously. Consequently, T3SS expression by a subset of the population may be sufficient to induce host cell disruption and nutrient release, while limiting the metabolic cost associated with producing this energetically expensive and complex secretion apparatus. Notably, the proportion of T3SS-expressing cells is strongly influenced by relative humidity: under high humidity (100%), up to 80% of *E. amylovora* cells express T3SS genes, whereas this proportion decreases to approximately 40% at 60% relative humidity [104].

### Phevamine a biosynthetic genes hsvABC

Alongside the *hrp* genes, the *hrp*-T3SS cluster of *E. amylovora* contains five open reading frames, organized in two operons, that encode proteins with enzymatic activity and whose genes are similarly dependent on HrpL for transcriptional activation [63,108]. Three of these genes, designated as *hsvA, hsvB*, and *hsvC* (*hrp-*associated systemic virulence A, B, and C) were shown to be required for full virulence and systemic spread in apple shoots [108], suggesting that they play an important role during infection likely by synthesizing a virulence-associated metabolite essential for host colonization. *hsvA* shares amino acid sequence similarities with amidinotransferase family proteins, which are known to be involved in the biosynthesis of bacterial toxins, such as phaseolotoxin and cylindrospermopsin [109,110]. Moreover, studies on the homologous operon from *P. syringae* pv. *tomato* demonstrated that the enzymes encoded by the *hsv* operon synthesize phevamine A, a small-molecule virulence factor that interferes with plant immune responses by impairing the generation of PAMP-induced ROS mediated by polyamines, such as spermidine and putrescine [111].

### Exopolysaccharides

*E. amylovora* produces three exopolysaccharides (EPSs), amylovoran, levan, and cellulose. The presence of a capsule associated with only virulent *E. amylovora* cells and not found on avirulent cells was first demonstrated by Bennett and Billing in 1978 [112], and the capsule was later shown to consist of amylovoran [113]. Amylovoran is a heteropolymer composed of galactose, glucose, and pyruvate residues arranged in branched repeating units [114] and is structurally similar to stewartan, an EPS produced by the related plant pathogen *Pantoea stewartii* subsp. *stewartii* [115]. Amylovoran biosynthesis is encoded by the 14-genes *ams* operon [116], and mutant strains unable to produce amylovoran are completely nonpathogenic. The quantity of amylovoran produced correlates with virulence, as low-producing strains exhibit significantly reduced virulence [117].

Levan is a fructose homopolymer synthesized from sucrose by a secreted levansucrase enzyme [118]. The gene encoding this enzyme, *lsc*, was identified by transposon mutagenesis [119], and *lsc* mutants displayed reduced virulence and impaired systemic movement in inoculated pear seedlings [119]. Phylogenetic analyses revealed that *E. amylovora* levansucrase was most closely related to that of the plant pathogen *P. syringae* and was characterized as a type-I levansucrase along with similar enzymes produced by *Zymomonas mobilis*, which are utilized in biotechnological applications [120].

Cellulose, a polymer of β-1,4-linked glucose residues arranged into fibrils, is a widespread bacterial virulence factor and a major structural component of biofilms [121]. Cellulose fibrils are the primary structural component of plant cell walls and have a tensile strength similar to steel [122]. Cellulose biosynthesis is widespread in bacteria and is a virulence factor in many human and plant pathogens [123]. *Erwinia amylovora* synthesizes cellulose and encodes six *bcs* genes whose organization is similar to that of other plant pathogens including *Pantoea ananatis* and *Dickeya dadantii* [123]. Cellulose production by *E. amylovora* was identified as a virulence factor and an important component of biofilms (see below), and cellulose fibrils linking cells in a network were visualized using scanning electron microscopy [124].

The EPSs produced by *E. amylovora* function separately and collectively in promoting virulence. However, there is still much to be learned concerning the role of EPSs during infection and systemic movement of *E. amylovora* through host plants. Amylovoran mutants are nonpathogenic and do not grow in shoot blight and immature pear fruit infection models [125,126]. To date though, the reason why amylovoran-deficient mutants are nonpathogenic has not been fully elucidated. It has long been thought that the amylovoran capsule protects invading *E. amylovora* cells from exposure to host defense compounds potentially by shielding elicitors of host defense that could induce PTI [127,128]. However, to our knowledge, a precise role for amylovoran in host immunity evasion has not been demonstrated.

### Biofilm formation in host xylem vessels

In addition to colonizing the cortical parenchyma, transmission electron microscopy studies showed that *E. amylovora* also inhabits xylem vessels in leaf petioles from infected shoots [129,130]. Subsequent scanning electron microscopy analyses revealed hallmark features of biofilm formation of *E. amylovora* cells in xylem, including initial attachment of *E. amylovora* cells to the inner wall of xylem vessels, localized growth originating from attached cells, production of a fibrillar extracellular matrix that spans the vessel lumen, and densely packed xylem vessels filled with cells embedded in the fibrillar matrix [34]. Biofilm formation in host xylem vessels is a common virulence trait in several Gram-negative plant pathogens, including *Ralstonia solanacearum* and *Xylella fastidiosa*, and is ecologically important to growth in this low-nutrient environment and also to maintain colonization as the interior of xylem elements is a high water-flow environment [131,132]. Attachment of cells to a surface is the critical step of biofilm formation [133], and *E. amylovora* attachment structures were identified and visualized [126,131]. Consistent with this requirement, an *E. amylovora* mutant that was completely devoid of attachment capability was determined to be unable to form biofilms [134]. Genetic analyses of *E. amylovora* biofilms demonstrated that the amylovoran EPS is essential and that levan contributes to biofilm formation [34]. More recently, the discovery of cellulose production by *E. amylovora* was accompanied by mutagenesis studies determining that cellulose contributes to the three-dimensional architecture of biofilms *in vitro* and is a main structural component of biofilms in leaf xylem [124].

### Functional roles for *E. amylovora* EPSs

**Figure 6.**
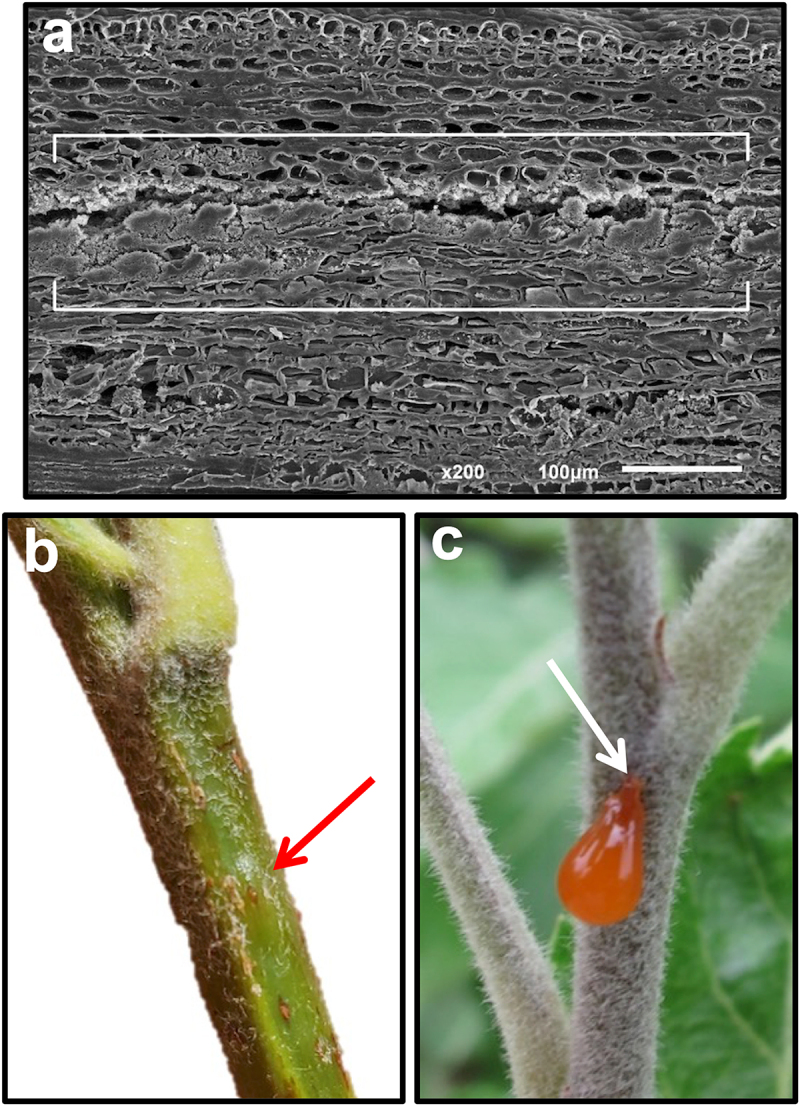
(a) Scanning electron micrograph showing a schizogenous cavity formed during the longitudinal migration of *Erwinia amylovora* cells in an infected branch (used with permission from [dougherty et al 2025]. (b) Erumpent mound on a “Gala” apple branch, site of internal pressure buildup of *E. amylovora* cells prior to generating a wound enabling bacterial ooze to exude onto the surface. (c) Exudation of an *E. amylovora* ooze droplet onto the surface of a “Jonathan” apple branch. The arrow denotes the site of the wound the ooze droplet (ca. 10 µL) has exuded from.

In the apoplast, amylovoran and levan are required for sliding motility, a phenotype that enables nonflagella-based cellular motility [135]. Since *E. amylovora* cells are known to lose flagellar motility in the apoplast [136], sliding motility provides a mechanistic basis for movement in the apoplast, which is hydrated due to the cell pore forming ability of DspE. The second function of *E. amylovora* EPSs involves facilitating pathogenesis and enabling the systemic spread of cells via intercellular spaces in the cortical parenchyma of branches and flower pedicels. The cortical parenchyma is the layer of living cells directly below the epidermis. *Erwinia amylovora* cells infect cortical parenchyma cells through actions of the T3SS which results in a hydrated apoplast that also provides nutrients to invading cells. As these cells continue to divide and fill the available space in the apoplast, further multiplication creates pressure termed by Schouten as multiplication pressure [137]. This increase in pressure resulting from increased bacterial biomass and swelling EPS ultimately results in an elevation of internal pressure that the plant cells cannot withstand, which leads to the formation of schizogenous cavities [137]. Histopathology studies have shown *E. amylovora* cells located in long longitudinally oriented cavities in stem parenchyma tissue that are generated by the multiplication pressure (Figure 6(a)). This pressure can also extend radially outward ultimately leading to the formation of erumpent mounds (Figure 6(b)) that cause a wound in the epidermis and the spilling out of an ooze droplet to the stem surface [12] (Figure 6(c)). As the ooze droplet emerges onto the plant surface, the force of gravity pulls the droplet downward, but the viscosity of the droplet and the EPS matrix prevents the ooze droplet from rolling off the plant surface (Figure 6(c)). Thus, EPSs contribute to the movement of *E. amylovora* in the apoplast by sliding motility, to the pressure mediated creation of a longitudinal movement pathway, and also to the egress of cells in ooze droplets which facilitates spread to new infection sites on the same tree or on other trees.

The discovery of *E. amylovora* cells in host xylem vessels, the characterization of *E. amylovora* biofilms in xylem, and the wilt-like symptoms of shoot blight have led some researchers to postulate that *E. amylovora* is moving through plants via the xylem and that fire blight is a vascular wilt disease. However, as elegantly discussed by Billing [138], movement of *E. amylovora* cells into mature xylem vessels in woody tissue of older branches would leave them with no means of escape from these vessels, and thus these cells would be “stuck” in the host. In contrast, there is direct microscopic evidence of *E. amylovora* cell masses escaping from ruptured xylem vessels in leaf petioles [139]. These immature vessels are flexible and have not been completely lignified. Thus, it is possible that leaves and leaf petioles are the predominant location of biofilm formation which enables biofilm development followed by the escape of cells following vessel rupture. Movement through stems then would predominantly occur via cortical parenchyma (summarized in [138]) that can enable *E. amylovora* cells to emerge from the host in ooze droplets [12]. Canker formation also occurs in these layers and gives *E. amylovora* cells surface access the following spring.

## Molecular regulation of virulence

**Figure 7.**
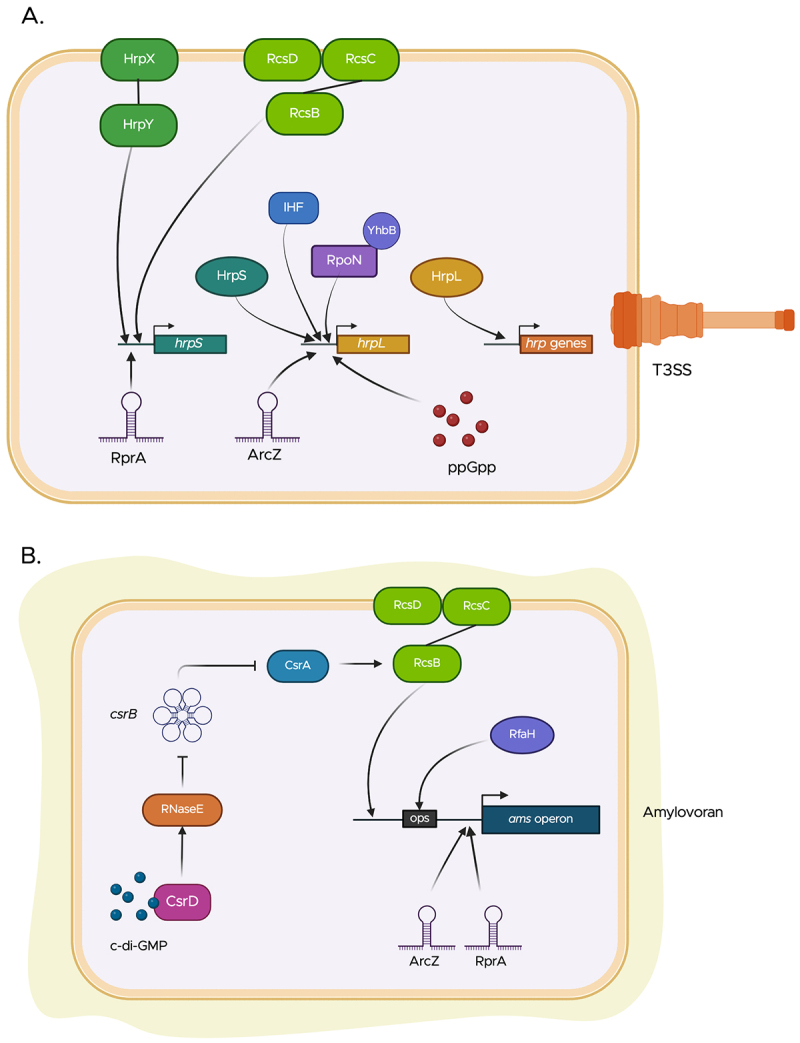
Multifactorial regulatory networks controlling the activation of pathogenicity determinants in *E. amylovora*. (a) The activation of the *hrp*-T3SS depends on the alternate sigma factor HrpL, whose expression is tightly regulated by multiple regulatory elements. These include the TCS HrpX/HrpY, the Rcs phosphorelay system, and the small regulatory RNA RprA which promote the expression of the enhancer-binding protein HrpS. In conjunction with integration host factor (IHF), RpoN and its modulator YhbB, HrpS upregulates the expression of the *hrpL* gene. The small regulatory RNA ArcZ, along with the alarmone secondary messenger (p)ppgpp, further promotes the upregulation of *hrpL*. (b) Amylovoran biosynthesis is activated by c-di-GMP through its binding to CsrD, together with RNaseE, promotes the degradation of *csrB*. This relieves repression of CsrA, allowing the upregulation of the *ams* operon by CsrA via the Rcs phosphorelay system. In addition, the transcription antiterminator RfaH binds upstream of the *ams* promoter and enhances the expression of genes within the *ams* operon. Figure created in https://biorender.com.

After detecting environmental and host-derived cues, *E. amylovora* modulates the expression of key pathogenicity and virulence determinants during different infection stages. This regulation is coordinated through many complex and tightly controlled multifactorial networks of regulatory elements, whose functions and activities are finely tuned to control the timing and amplitude of the regulatory response. These elements include two-component signal transduction systems, small noncoding RNAs, and the nucleotide messenger molecules cyclic di-GMP, and (p)ppGpp [140]. Summaries of regulation of the T3SS and of amylovoran biosynthesis are shown in Figure 7(a,b).

### Two-component systems

Two-component systems (TCS) are widespread signal transduction pathways found in both prokaryotes and eukaryotes, which enable organisms to deploy adaptive responses, mainly through the regulation of gene expression, after sensing a wide variety of input stimuli (reviewed in [141,142]). TCSs are typically composed of a membrane-bound sensor that exhibits a histidine kinase activity and a cytoplasmic transcriptional regulator. Upon perception of an external cue through its extracytoplasmic domain, the histidine kinase autophosphorylates at a conserved histidine residue. The phosphoryl group is then transferred onto a specific aspartate residue in the corresponding response regulator, activating downstream signaling pathways (reviewed in [141,142]). This mechanism allows bacteria to rapidly modulate gene expression in response to changing environmental and host-associated signals. In *E. amylovora*, the expression of structural components of the *hrp*-T3SS, effector proteins, and the additional members of the HrpL regulation is modulated by the TCSs HrpX/HrpY along with the σ^54^-dependent binding protein HrpS, the integration host factor (IHF), RpoN (σ^54^), and its modulator protein YhbH [143–147]. Under T3SS-inducing conditions, such as low nutrient availability and acidic pH, HrpX autophosphorylates, and subsequently activates the response regulator HrpY, which in turn enhances the expression of *hrpS*. This allows the initiation of σ^54^-dependent transcription of *hrpL* [143,145,147].

The Rcs phosphorelay transduction system is a nonorthodox TCS present in several members of the Enterobacteriaceae family. This system is typically composed of three core proteins: RcsC, a transmembrane hybrid histidine kinase; RcsD, a transmembrane phosphotransfer protein; and RscB, a response regulator. In addition, several additional auxiliary proteins have been shown to be involved in this signaling pathway (reviewed in [148,149]). In *E. amylovora*, gene regulation by the Rcs phosphorelay plays a key role in pathogenesis, as single and double mutations of the *rcs* genes result in loss of pathogenicity in immature pear fruit [150]. The Rcs phosphorelay regulates the T3SS, as the expression of *hrpS* is activated by RcsB, in a HrpX/HrpY-independent fashion [145,150]. Similarly, RscB is essential for the activation of amylovoran biosynthesis and acts as a positive transcriptional regulator of the *ams* operon through binding in the promoter region of *amsG* [151,152]. In addition, the Rcs phosphorelay system represses motility by downregulating the expression of the genes encoding the master regulator complex of flagellar motility, FlhDC [150,152].

The GrrS/GssA (GacS/GacS) and EnvZ/OmpR TCSs have been described as global regulators that coordinate bacterial gene expression in response to different environmental conditions, such as changes in extracellular osmolarity and temperature shifts [153,154]. Both GrrS/GrrA and EnvZ/OmpR systems have been shown to negatively regulate the expression of T3SS genes and amylovoran biosynthetic genes in *E. amylovora*, as evidenced by a significant increase in the expression of *hrpL*, *hrpN* and *dspE* as well as a significantly higher activity of the *amsG* promoter exhibited by single and double mutants of *envZ*, *ompR*, *grrS*, and *grrA*, when compared with the wild-type strain [155]. In addition to their co-regulatory roles, these systems antagonistically regulate motility. While EnvZ/OmpR has a positive impact on motility, GrrA/GrrS has been shown to act as a negative regulator [155].

### Small noncoding RNAs

Another strategy that enables bacteria to rapidly adapt to diverse environments is through the expression of small noncoding RNAs (sRNAs), which typically function at the post-transcriptional level, modulating the translation and transcript stability of their targets or by binding and/or sequestering other regulatory elements (reviewed in [156,157]). sRNAs constitute a major layer of regulation in *E. amylovora*, which is integrated into broader regulatory networks.

#### csrB

*csrB*, a widely studied and well-characterized regulatory sRNA among eubacteria [158], has been similarly identified as a central regulatory element for *E. amylovora*. *csrB* functions as a molecular sponge that binds and sequesters the RNA-binding protein CsrA, which is responsible for the post-transcriptional activation and repression of numerous target mRNAs [158]. In *E. amylovora, csrB* has been demonstrated to downregulate the expression of T3SS-related genes through its antagonistic action on CsrA. Likewise, *csrB* represses the expression of *ams* genes, which results in a significant decrease in amylovoran biosynthesis, and *csrB* has a negative impact on bacterial motility [159,160]. Additionally, the expression of *csrB* is positively regulated by the GrrS/GrrA TCS and IHF, thereby affecting CsrA downstream functions [159,160].

#### Hfq and Hfq-regulated sRNAs

The chaperone protein Hfq is a ubiquitous RNA-binding protein that, through limited base pairing, stabilizes sRNAs and facilitates their interactions with target mRNAs, leading to the subsequent molecular regulation of these targets. Through Hfq, bacteria are able to modulate a plethora of processes at a post-transcriptional level by mediating the binding of its associated sRNAs to the 5’ untranslated region (UTR) of target mRNAs rendering this region inaccessible for translation initiation, or breaking secondary structures that prevent ribosomal binding to enable translation (reviewed in [161]). An *E. amylovora* Δ*hfq* mutant was significantly reduced in virulence in both the immature pear and shoot blight models of infection, translocation of the major T3 effector DspE was reduced by 66% and amylovoran production was reduced by 50% [162]. Further phenotypic evaluation of the *E. amylovora* Δ*hfq* mutant revealed that critical virulence determinants including biofilm formation and motility were significantly impacted [162]. This regulation might be direct, through direct interaction of an Hfq-dependent sRNA(s) with the mRNA(s) encoding virulence factors or through post-transcriptional regulation of other regulatory elements that control the expression of virulence factors. Transcriptomic analyses revealed the presence of 40 candidate Hfq-dependent sRNAs in the genome of *E. amylovora*, several of which regulate virulence-associated traits [163]. Among these, ArcZ, OmrAB, and RmaA are involved in the regulation of flagellar motility in *E. amylovora*, by modulating transcriptionally and post-transcriptionally, the expression of the master flagellum regulator *flhDC* [164]. RmaA and ArcZ indirectly control the transcription of *flhD* via interaction with additional regulatory proteins, such as the leucine-responsive protein (Lrp). Moreover, ArcZ was demonstrated to directly bind to the 5’UTR of the *flhDC* mRNA to repress translation. Likewise, OmrAB is proposed to regulate *flhDC* at a post-transcriptional level, likely by direct interaction with its 5’UTR [164,165].

Phenotypic analyses have further demonstrated that the sRNAs OmrAB and RmaA (also known as Hrs6) act as negative regulators of amylovoran biosynthesis, while ArcZ and RprA exert positive regulation in this process [163,166]. These two sRNAs play important roles in coordinating the different stages of biofilm development. Whereas ArcZ negatively regulates initial attachment to solid surfaces during the early stages of biofilm formation, RprA negatively modulates the biosynthesis of levan and cellulose, while activating flagellar-dependent motility, promoting the dispersal of cells from mature biofilms [163,166]. Interestingly, Peng and collaborators [166] also demonstrated that the expression of RprA is controlled by the Rcs phosphorelay system.

### Cyclic di-GMP (c-di-GMP) and (p)ppGpp

C-di-GMP is a ubiquitous secondary messenger molecule found in all major bacterial phyla, demonstrated to be involved in the coordination of the transition from motility to sessility, through the regulation of flagellar motility, EPS production, and synthesis of attachment structures. Furthermore, numerous additional processes have been demonstrated to be regulated by this diffusible intracellular molecule, allowing bacteria to adapt to numerous environmental niches (reviewed in [167]). C-di-GMP is synthesized by diguanylate cyclases (DGC), containing a characteristic GGDEF domain, from two GTP molecules, and it is hydrolyzed by phosphodiesterases (PDE), with an EAL or HD-GYP catalytic domain, into GMP or linear 5′-phosphoguanylyl-(3′–5′)-guanosine (pGpG). Intracellular c-di-GMP interacts with a variety of receptor proteins, modulating their regulatory roles on specific targets [168]. The genome of *E. amylovora* encodes five functional DCGs and three PDEs, along with four additional proteins containing nonfunctional GGDEF and/or PDE domains. Genetic analyses using site-directed mutagenesis and gene overexpression revealed that c-di-GMP is a negative regulator of virulence through the downregulation of the expression of the T3SS master regulator HrpL [169,170]. Similarly, *E. amylovora* employs c-di-GMP to negatively regulate flagellar motility.

Elevated intracellular c-di-GMP levels in *E. amylovora* result in the stimulation of biofilm formation on solid surfaces [169]. This is the result of the activation of multiple key components of *E. amylovora* biofilms by the second messenger. Kharadi and Sundin [171] demonstrated that c-di-GMP contributes to the activation of amylovoran biosynthesis through the Csr regulatory pathway by binding to CsrD, a RNaseE specificity factor that contains nonfunctional GGDEF and EAL domains. Binding of c-di-GMP to CsrD promotes the degradation of *csrB*, increasing the pool of free CsrA available to activate the transcription of the *ams* operon [171]. This regulation is indirect and is thought to occur via CsrA binding to and increasing the translation of RcsB [160]. Under low c-di-GMP levels (i.e. T3SS active in apoplast), CsrA is bound by the molecular sponge CsrB which reduces the transcription levels of ams genes [160]. Increasing concentrations of c-di-GMP also stimulates the biosynthesis of cellulose via allosteric binding to the PilZ domain in the cellulose catalytic subunit BcsA [124]. More recently, findings by Yuan and collaborators [172] demonstrated that the sRNA-binding protein ProQ contributes to the degradation of intracellular c-di-GMP by activating the expression of two PDE-encoding genes, which results in a negative impact on the biosynthesis of cellulose in *E. amylovora*.

The stringent response, which coordinates bacterial adaptation to nutritional starvation and various environmental stresses, relies on the linear nucleotides guanosine tetraphosphate ppGpp and guanosine pentaphosphate pppGpp, collectively known as (p)ppGpp or alarmones. Elevated (p)ppGpp intracellular levels trigger global metabolic changes due to the reprogramming of bacterial gene expression (reviewed in [173]). These secondary messengers are synthesized and degraded by members of the RelA/SpoT homolog (RSH) family of proteins. RelA synthesizes (p)ppGpp from ATP and GTP in response to amino acid limitation, while SpoT exhibits a dual catalytic role of synthesis and hydrolysis of these alarmones under carbon-source starvation conditions. (p)ppGpp, along with RpoN and the RNA polymerase-binding protein DskA, act synergistically to modulate global transcriptomic reprogramming to transition from an active cellular growth stage to a survival-oriented state [174,175]. In *E. amylovora* (p)ppGpp accumulation elicits the activation of T3SS, in nutrient-limited conditions, such as those encountered in the apoplast, as simultaneous mutation of *relA* and *spoT* genes, as well as mutation of *dskA*, renders the bacterium nonpathogenic and leads to a significant downregulation of *hrp-*T3SS-associated genes, such as *hrpL*, *hrpA*, *dspE,* and *hrpN* [176]. Furthermore, transcriptomic analyses further showed that (p)ppGpp positively influences the expression of genes associated with amylovoran and levan biosynthesis, as well as genes encoding the structural components of the flagellum in *E. amylovora* [177].

### Additional regulators of amylovoran production

The 12-gene amylovoran operon (*amsGHIABCDEFJKL*) is 15.8 kb in length with an unusually large 740 bp upstream noncoding sequence. As described above, known regulators of the *ams* operon include the conserved Rcs phosphorelay, CsrA and *csrB*, cyclic di-GMP via CsrD, and the GrrS/GrrA and EnvZ/OmpR two component systems. The RcsA and RcsB conserved regulatory proteins have been shown to bind to a region from −578 to −501 upstream of the translational initiation codon of *amsG* [151]. In addition, a conserved *ops* (operon polarity suppressor regulatory element) is located from −237 to −226 upstream of the *amsG* start codon. The *ops* site is bound by RfaH, a transcriptional antiterminator that reduces pausing in the production of long transcripts [178]. Klee et al. [179] elegantly demonstrated the effect of a Δ*rfaH* mutant on the expression of each of the 12 genes in the *ams* operon. While the expression of the first two genes *amsG* and *amsH* was reduced approximately twofold in the Δ*rfaH* mutant, distally located genes including *amsD*, *amsE*, *amsF*, and *amsJ* exhibited greater than a 10-fold reduction in expression compared to the wild type [179].

Other regulators of amylovoran biosynthesis are known; however, the regulatory mechanisms are unclear. Lon protease, a conserved cytosolic protease in bacteria, is a negative regulator as mutation of *lon* leads to the overproduction of amylovoran [180]. Similarly, mutation of *hns* encoding a conserved histone-lie protein results in an increase in amylovoran production [181]. Finally, AmyR, an ortholog of *E. coli* YbjN, also negatively regulates amylovoran production by an unknown mechanism [182]. These regulators might be functioning as a check on amylovoran biosynthesis as an energy conservation mechanism for cellular fitness. Indeed, Yuan et al. [183] recently demonstrated that a complete functioning thiamine biosynthesis pathway is required for amylovoran biosynthesis and that thiamine enhances bacterial respiration that provides the energy requirements needed to produce this EPS.

## Future view of fire blight management

The focus of fire blight management strategies has been to limit infection of flowers leading to blossom blight symptoms. This is a sound strategy because flowers are short lived and need only to be protected over a typical 7–14-day time span and *E. amylovora* cells are located on stigma surfaces and so are accessible to materials applied by spraying. However, this short time frame is also counterbalanced by the ability of rapid pathogen growth on stigmas during conducive weather and by the spreading of pathogen cells between flowers by pollinating insects.

The susceptibility of commercial apple and pear cultivars has driven the need for highly effective materials used for blossom blight management because once infection occurs, *E. amylovora* bacteria continue to amplify populations that are now systemic inside hosts, systemic spread can ultimately result in tree death, and during this systemic spread cells can be partitioned to ooze droplets facilitating disease spread to new trees [12]. To date, the antibiotic streptomycin has been the best bactericide for blossom blight management due to its rapid killing of *E. amylovora* cells on flowers and because it is partially systemic and can penetrate into the floral cup to kill cells that have already started to infect flowers [184,185]. However, streptomycin resistance is a widespread problem due to reliance on this material in commercial orchards since the 1970s [186,187].

### Host resistance

Plants have evolved highly sophisticated surveillance systems functioning in the detection of invading pathogens and the activation of immune responses. The deployment of plant immune responses in response to microorganisms operates through sequential and multilayer processes shaped by long-term evolutionary interactions between plants and microbes that challenge them. The first layer of innate immune responses is induced upon the perception of conserved pathogen or microbial-associated molecular patterns (PAMPS/MAMPS), via surface receptors in the plant cell known as PAMP-recognition receptors (PRR). This defense mechanism is referred to as PAMP-triggered immunity (PTI) and constitutes a baseline of disease resistance [188,189]. Successful pathogens in turn deliver effector proteins into the host cell that suppress PTI, resulting in effector-triggered susceptibility (ETS). Some of these effectors are specifically recognized by cognate plant resistance (R) proteins, usually belonging to the nucleotide-binding/leucine-rich repeat (NLR) family of receptors, through direct gene-for-gene interactions. This initiates a second and more robust layer of immunity signaling termed effector-triggered immunity (ETI), resulting in a resistant phenotype.

The use of host resistance is a critical management strategy for crop plants, such as rice, where many resistance genes are available that confer an ETI response to the pathogen *Xanthomonas oryzae* [190]. In apples, the prospects for incorporating host resistance are much more difficult. First, apples are sold by cultivar and popular cultivars, such as “Gala” and “Fuji,” appeal to consumer preferences of sweetness and crispiness. Unfortunately, nearly all of the best-selling apple cultivars are highly susceptible to fire blight. However, this has not stopped research into host–pathogen interactions and the search for resistance genes in wild *Malus* spp.

To our knowledge, ETI is not known in modern commercial apple cultivars descendants of *Malus sierversii* [29]. However, during the past 15 y, ETI has been observed in wild *Malus* species recognizing two different *E. amylovora* T3 effectors. AvrRpt2_Ea_ is recognized by the resistance gene FB_Mr5 of the fire blight-resistant crabapple species *Malus* × *robusta* 5 (Mr5) through a gene-for-gene relationship [191]. Prokchorchik and collaborators [192] further demonstrated that the recognition of AvrRpt2_Ea_ in Mr5 requires the proteolytic processing of MdRIN4 (*M. domestica* RPM1-interacting protein 4), a decoy protein whose cleavage is detected by the FB_Mr5 resistance gene. This resistance mechanism parallels the recognition of AvrRpt2 from *P. syringae* by the RPT2 resistance gene after RIN4 processing in the *Arabidopsis thaliana* plant model [84,86]. Unfortunately, this resistance can be broken down by a single nucleotide polymorphism (SNP) that results in a C156S amino acid substitution in AvrRpt2_Ea_ [191].

Additional studies by Wohner et al. [193] suggested a gene-for-gene relationship between *eop1* and the resistant ornamental cultivar *Malus* “Evereste” as well as the wild species *Malus floribunda* 821, as deletion of *eop1* resulted in significantly greater symptom development in these hosts compared with those produced by the wild type strain. More recently, a similar gene-for-gene interaction was reported between Eop1 and the resistance locus FB_Mar12 from *Malus* × *arnoldiana* [194] and a potential new source of resistance was discovered in pear cultivars “Conference” and “Harrow Sweet” [195].

In addition to resistance (*R*) gene-mediated defense, which relies on the introduction of resistance genes into the host, susceptibility (*S*) gene-based resistance – achieved through silencing or removal of host susceptibility genes – is considered a more durable strategy for disease control. The HrpN-interacting protein from *Malus* spp. (HIPM) is a transmembrane receptor in apple that interacts with the type III secreted harpin HrpN from *E. amylovora* and is required for successful infection, identifying HIPM as a susceptibility gene [196]. HIPM is constitutively expressed in apple tissues. Campa et al. demonstrated that silencing HIPM in transgenic apple significantly reduced susceptibility to *E. amylovora* [197]. Further studies revealed that HIPM interacts with an oxygen-evolving enhancer-like protein from apple (MdOEE), a protein potentially involved in the oxidative burst required for infection, highlighting MdOEE as a novel potential target for disease-resistant apple breeding [197].

Breeding for resistance in tree fruit crops, such as apple, is a very long-term process because the hosts containing the resistance gene typically produce small, bitter, poor-quality fruit, and many crosses are needed to integrate resistance genes into a cultivar with excellent fruit quality. In addition, incorporating multiple-resistance genes into a single cultivar is optimal because of the chances for pathogen mutations disrupting the ETI response. Current research in this arena involves using technologies such as incorporating early flowering traits into recipients to speed up the process of selection after mating experiments [198]. However, breeding for resistance to fire blight remains a long-term endeavor.

### Antibiotic alternatives for blossom blight management

Significant research has been done evaluating alternatives to antibiotics for blossom blight management. Much of this work is directed toward strategies that suppress *E. amylovora* growth on stigmas or induce host resistance as bactericidal alternatives are less readily available for deployment in plant agriculture. During the past 3 y, several strategies have been tested to develop potential new fire blight management materials including photodynamic therapy, antivirulence compounds, and compounds that induce resistance in the host. Photodynamic therapy using light-activated Mg chlorophyllin or titanium oxide to generate ROS was evaluated in the field with control not significantly different from an antibiotic standard in three of four experiments or two of two experiments, respectively [199,200]. A third photodynamic therapy at the experimental stage involves the use of ZnO quantum dots [201]. The use of ultraviolet radiation to irradiate flowers at night with a dose of 200 J m^−2^ using a portable apparatus that moves over trees was highly effective with control not different from streptomycin over 2 y of field trials [202]. Examination of the T3SS inhibitor TS108 was done in field studies over 7 y at four locations with control not significantly different from an antibiotic standard in three of five tests [203]. A second antivirulence compound tizoxanide is in testing at the experimental stage [204]. The isolation and characterization of bacteriophage for blossom blight management has been intensively studied in the United States, Europe, and Korea [205–208]. Results have been variable illustrating the need for more in-depth research and more field research to identify better methods to deploy phage to protect them from environmental stresses as well as better position them to function on stigmas [209].

Conditioning trees for management by applying materials that stimulate plant defense has worked particularly well with shoot blight management. Prohexadione-calcium (ProCa), a gibberellin synthesis inhibitor that inhibits tree growth, was shown to reduce shoot blight infection by causing cell wall thickening of plant parenchyma cells to widths that were larger than the length of the *E. amylovora* type III pilus [210]. More recently, ProCa was also shown to induce the expression of some marker genes for systemic acquired resistance (SAR) [211]. The combination of ProCa + ASM was shown to be an excellent inducer of SAR and showed excellent efficacy in the field [211–214]. The use of lower rate combinations of ProCa + ASM was also effective in high-density plantings and had some positive benefits on fruit size and quality [215].

Finally, knowledge of the apple and pear flower microbiome may ultimately inform new disease management tactics or supplement existing management strategies. Members of the flower microbiome have documented roles in suppressing *E. amylovora* growth, affecting *E. amylovora* behavior, altering environmental pH, and inducing host defense responses [216–218]. Studies examining how the microbiome is assembled, disseminated, and maintained will help guide the selection of microbial members that are most resilient and dominant on flowers [2,21,219,220]. Among them, the yeast *Aureobasidium pullulans* has been demonstrated with great promise in suppressing fire blight [218]. Additional studies on how the microbiome is manipulated by potential biocontrols will also inform deployment strategies [206,221–223]. Future work is needed to identify more biocontrol candidates that provide consistent, high levels of protection against fire blight, either alone or in combination with other management materials [224,225].

## Perspectives and future studies

Significant progress has been made in understanding the biology of *E. amylovora*, the disease cycle of fire blight, the genes involved in pathogenesis and how those genes are regulated. However, even with this tremendous progress, fire blight remains extremely difficult to manage. A major issue impacting management is the high disease susceptibility of customer-favored apple cultivars. Besides trying to address this with host resistance, a better understanding of host susceptibility and marker-assisted selection against those genes could be very useful in breeding.

Improved understanding of the *E. amylovora* pathogen can impact novel disease management strategies. For example, the systemic migration of *E. amylovora* cells via the apoplast in the cortical parenchyma layer of cells explains why determinants, such as phevamine A, are required for migration through infected shoots as this is a mechanism to suppress active host defense that functions in the apoplast. A focus on systemic migration through cortical parenchyma would be a positive step toward continuing to study the role of eliciting host defense responses in developing novel management strategies for fire blight. Ultimately, continued study and improvement of novel management tactics under field conditions is required to generate potential sustainable solutions to this devastating disease.

## Data Availability

Data sharing is not applicable to this article as no new data were created or analyzed in this study.
